# An Inquiry into the Nature and Properties of the Blood, as Existent in Health and Disease

**Published:** 1819-10-01

**Authors:** 


*An Inquiry into the Nature and Properties of the Blood, as existent in Health and Disease.*

By C. TURNER THACKRAH, M. Roy. Col. Surg. and Lec. Soc. Aph. 1 vol. 8vo. 132 pages. London, 1819.

" Est quodam prodire tenus, si non datur ultra." HOR.

OF the principles and laws of the animal economy, as Mr. Thackrah observes, few are more intricate than those which regard the blood. The nature of the subject, therefore, admits of ample scope, not only for experimental research, but for unbounded speculation, contradictory notions, and inconclusive statements. Still, we are not to entertain the opinion that investigation is fruitless, nor abandon the hope that truth is ultimately discoverable. In the present state of physiology, *data* are greatly wanted, and conclusions cannot be drawn with any thing like mathematical certainty, but turn on the balance of probability or improbability. He, therefore, who adds to the stock of *accurate data*, is a benefactor to medical science, by contributing to the substratum on which the whole fabric must eventually rest.

The work before us is the improved publication of a manuscript Essay on the subject, which gained a prize, offered by Mr. Astley Cooper, for the best Dissertation on the Blood. This, of itself, is a strong recommendation; but the work contains more intrinsic proofs of merit than any medal can bestow.

"The practical parts of this Essay require little comment. Extensive opportunities have been afforded me of examining the blood in disease; and of these I have gladly availed myself. To state what observation and experiment have taught me, has been my principal object; not to form a compilation from the works of others, nor to canvas the justice of their notions, or the correctness of their remarks."

In the first Chapter Mr. Thackrah, among other physiological subjects, discusses the famous Hunterian opinion respecting the vitality of the blood; which, by the following quotation from Hippocrates, appears, like many other opinions, to be of no very recent formation.

"Ηγεομαι δε εμπροσθεν μηδεν ειναι μαλλον των εν τω αωματι ξυνβαλλομενονες φρονησιν αν, η το αιμα. Τȣτο δε οταν εν τω καθεςτηκοτι σχηματι μενη, μενει και η φρονησις, εξαλλασσοντος δε τȣ αιματος, μεταπιπτει και το φρονημα."—De Flatibus.

We might quote *Scripture*, indeed, for the blood being the "Life of the beast;" and Harvey, among others, clearly considers this fluid to possess life and *locomotion*.

"Habet [seil. sanguis.] profecto in se animam primo ac principaliter, non vegetativam modo, sed sensitivam etiam et motivam." *De Generatione.*

But notwithstanding the authority of Hippocrates, Harvey, and Hunter, there is great reason to question the truth of this opinion.

"Another difficulty attends Mr. Hunter's theory. If the blood possess life, where is this property first acquired? We know that he blood is formed from the digestion of various inanimate subt ances. "Is the chyle alive?" Mr. H. thinks it is. Admitting h e supposition, we must ask, "Is the *Chyme* endued with vitality, or does our food, by commixing with the salivary and gastric juices, acquire its living principle?"

"If in *vitality*, be implied an *independent power of motion*, we should expect that the circulation of the blood was spontaneous, and that its properties were of an active kind. But of such inherent faculty we have no evidence. We observe a simply passive motion of this fluid: the heart impels it, the vessels convey it, the glands act on it." P. 10, 11.

In respect to the *specific gravity* of blood, Mr. Thackrah's experiments, though marked by considerable diversity, lead him to estimate it, as compared with water, at 1050, water being 1000. The medium proportion of crassamentum to serum in health, is as 13 or 14 of the former to 10 of the latter.

We must pass over all that part of the work in which the chemical qualities of the blood are examined; but shall rest a little on the 4th Chapter, which treats of "the effect of certain circumstances in retarding or accelerating" the *coagulation* of this fluid.

A subject which has particularly engaged our author's attention is the "*comparative periods of coagulation, as influenced by the strength or weakness of the vascular action.*" This is an inquiry of considerable practical importance, as well as physiological interest. The experiments we cannot insert, but only the results.

"From these statements, then, it appears that in the dog, sheep, horse, and hog, *the blood coagulates slowly in regular proportion to the tonic state, or that condition of the system in which the vital powers are strongest.* The blood received immediately before the death of the animal, first assumes this change; next, that which is taken at the middle period; and lastly, that which is received on the first effusion from the wound." P. 48.

This statement is corroborated by the experiments of Hewson and others; and in bleeding the human subject, Mr. Thackrah says, the same rule will hold good, to wit, that the first flowing blood has the least disposition to coagulate. Thus, for instance, in Exp. xviii.

"From the arm of a female labouring under fever, blood was drawn to the amount of a pound and a half; a portion of which, received in a tea-cup on the *first effusion* remained fluid for *seven minutes*; a similar quantity taken immediately before tying up the arm, was caked in 3 min. 30 sec.

Besides these observations, many more might be adduced, if it were necessary, to prove that "*a state of diminished tone is most favourable to the concretion of the blood.*" A tendency to deliquium has also a considerable effect.

"When concretion has been noticed to take place in five minutes, the occurrence of faintness, I have remarked, has immediately effected such a change in the state of the blood, as to induce this process in two minutes, and when ninety seconds were required, deliquium has instantly changed the period to 40."

This is a fortunate circumstance; for, as the first check to bleeding is given by the formation of coagula on the mouths of the vessels, were this process *slow* instead of quick, in proportion to the degree of debility, every considerable hæmorrhage, especially uterine, would prove fatal.

The 5th Chapter, on "the cause of the blood's coagulation," is very interesting, and we regret that we cannot do justice to it in this place. From a great variety of experiments, most ingeniously devised and skilfully executed, Mr. Thackrah has come to the conclusion that the *fluidity* of the blood depends principally on the *vitality* of the containing vessels, and coagulation on their death.

"The inference drawn from the observations of this section is obvious. Experiments, in which the greatest attention was paid to accuracy in execution, and honesty in detail, have shewn that blood retained for the requisite period, is found fluid in a living vessel, partially or irregularly coagulated in a semi-living vessel, and firmly concreted in one devoid of vitality. I conclude, therefore, that *the vital or nervous influence is the source of the blood's fluidity, and its loss the cause of coagulation.* P. 79, 80.

CHAP. VI. Changes produced in the blood by disease.

This is a subject too little attended to in modern times. For, although but few indications can be safely drawn from the state of the blood *alone,* yet, in conjunction with all the symptoms and phenomena of disease, it becomes an important circumstance of attention. It is on this chapter, therefore, that we are inclined to dwell with some analytical minuteness.

We shall pass over Mr. Thackrah's observations on the quantity, colour, and temperature of morbid blood. Its *specific gravity,* from our author's experiments, is *diminished* in disease; but this diminution has not been proved to have any relation to the degree or character of the morbid action.

"The marked disparity between the periods of coagulation, when the system is under the influence of active inflammation, or remains unbroken by disease, and when the vital powers are reduced, clearly points out the importance of the subject in a curative view. Whoever pays attention to the circumstance, will, I

am persuaded, accede to the opinion, that *the speedy occurrence o concretion on the effusion of blood, affords a reason sufficiently cogent for the discontinuance of depletory measures.*" P. 90.

The firmness of the blood's coagulum has been considered a distinctive mark of the tonic state of the system—its great tenacity a characteristic of inflammation—its looseness a sure proof of debility. During inflammatory excitement the fibrine of the blood is known to be considerably augmented. Mr. Whiting found it compose $\frac{7}{1000}$, in acute rheumatism; whereas, in health, it made about $\frac{1}{1000}$ of the blood. The effects of this increase of fibrine on the coagulum are great, as might be expected, the crassamentum being larger, and much more dense in its texture.

"Reflection on this circumstance has generally a considerable effect in the treatment of disease; most practitioners thinking themselves authorized to repeat venesection, where the first-drawn blood has a firm crassamentum, and obliged to desist from the antiphlogistic regimen, when the coagulum is readily broken. These marks, however, ought not to be wholly relied on. In acute diseases, it is frequently found that the serum is slowly exuded; and hence, unless a due time elapse before examination, the coagulum is soft, from the serum it contains. Here, upon the general principle, the practitioner would desist from further evacuations, concluding the system to be greatly reduced. Sometimes, also, from the adhesion of the coagulum to the side of the vessel; from the kind of vessel, or other cause, the separation of the serum is prevented for many hours; yet, on the removal of such attachment, or on the division of the coagulum, the serum is effused, and the crassamentum becomes firm. The size of the vessels has also a considerable effect on the exudation of the serum, and consequently, on the density of the remaining coagulum. The fluid of blood received in a basin, is usually in greater proportion than that contained in a small cup; and, of course, the cake in the latter is looser than that of the former. If, however, on the *division of the coagulum, at the expiration of from eight to twenty-four hours, there ensue no considerable effusion of serum, and the crassamentum remain extraordinarily firm,* I believe that *further depletion is fully warranted.*" P. 95-6.

In acute maladies the coagulum is generally dense; nevertheless we frequently derive much benefit from bleeding, even when the crassamentum is soft and yielding; nor should we, in such cases, hesitate to repeat the depletion, provided other circumstances indicate its propriety. Dr. Watt, in his work on diabetes, remarks, that great advantage accrued from venesection, though the coagulum was loose and black; and that, on repeated evacuations of blood, the crassamentum became much firmer, and of a more natural hue. But it is by no means to be inferred that *inflammation* exists in every case where blood-letting proves beneficial.

The relative proportion of serum and crassamentum affords considerable evidence, Mr. Thackrah thinks, of the state of the system. He concludes, from experiments, that the serum is *usually* in a proportion *inverse* to the strength of the system. It is a curious fact that "*the serum is relatively increased during the continuance of bleeding*; and it is surprising how great a change in this respect the lapse of a minute produces." In this place Dr. Weatherhead suggested the following query.

"Does not the abundance of serum in the last drawn cup, arise from the immediate effect of the bleeding in rousing the energies of nature to absorb serum from the different cavities, and thus occasion the fact you have remarked. If this be the true cause, it will likewise account for the benefit derived from, and authorize the detraction of blood in both sanguineous and serous extravasations, wherein the strength has not thereby been materially diminished." P. 98.

We have not the least doubt of the truth of Dr. Weatherhead's hypothesis. Any evacuation from the system, as vomiting, purging, sweating, or bleeding, sets the absorbents to work, unless these evacuations be carried to excess, when a contrary effect seems to be produced.

We introduce the 51st experiment in illustration of this curious phenomenon.

"About one pound of blood was substracted from the arm of a muscular man, labouring under angina pectoris. It was weighed three days afterwards.

| | Ser. | Cras. | Ser. | Cras. |
|---|---|---|---|---|
| 1 Cup had | 160 | 360 or as | 10 | to 22·5 |
| 2 | 420 | 594 | 10 | 14·1 |
| 3 | 418 | 736 | 10 | 17·6. |

Great faintness occurred on filling the third cup." P. 99.

However the fact may be as to the proportion of the blood's constituents during hæmorrhage, certain it is, observes our author, that frequent bleeding diminishes the quantity of the crassamentum in the circulatory system; and equally certain that, in most diseases of the atonic character, and in a reduced state of the vital powers, the quantity of serum is large.

"It is by no means an unfrequent occurrence, however, for debility to be combined with acute disease. In this case, according to the opinion I have stated, are two principles or causes, opposite in their nature, and tending to produce contrary results, and in proportion as the one exceeds the other, shall we have a diversity in the quantity of serum." P. 102.

As manifesting the necessity of moderating the quantity of crassamentum in febrile and inflammatory states of the system, Mr. T. relates some cases in illustration. Thus in Exp. 58, " from the first attack of the complaint to the second venesection, the child was in great suffering; but no sooner was the proportion of the crassamentum reduced to its healthy degree, than the pain was greatly relieved, and the malady in twenty-four hours completely removed."

It might be objected here, however, that the disappearance of the crassamentum was rather the *effect* than the cause, of the reduction of vascular excitement.

Dr. Mills, of Dublin, in most of the cases of acute fever which he records, found the serum in very small proportion to the crassamentum. In some acute maladies, scarcely a drop of serum is exuded; and what is remarkable, a similar circumstance has been observed in *asthma*.

The coat of yellowish size which sometimes covers the surface of blood, has greatly engaged the attention of the medical world, and has usually been considered the criterion of inflammation, since it is commonly found in acute rheumatism, pleurisy, and other phlegmasiæ. It seems probable that a variety of circumstances tend to produce this fibrous tunic on the blood. It is well known to be pretty generally present in pregnancy, especially in the advanced stage. In cases where the mineral waters, and even the vegetable acids have been freely administered, this phenomenon has been observed—in fact, it will often exist when the system is under any actively morbid influence. Various causes have been assigned for the buff coat of the blood; but none of them are free from objections. The following is our author's solution.

" On drawing blood during high vascular action, I had repeatedly noticed, that the first cup, which remained fluid the longest had the strong size—the surface of the second had transparent spots, and the third, which was soonest concrete, had no such appearance. Hence I was led to infer, that the formation of the buff-coat in such diseases depends on the blood's indisposition to coagulate, such tardiness of concretion allowing the red particles to subside, P. 1 11

Mr. Thackrah is inclined to believe that the vital energy is preternaturally excited in active disease, and thus, by its effects on the blood vessels, protracts the period of coagulation.

"The practical conclusions, which this subject induces, demand serious attention. The fibrous tunic is admitted to present itself in cases of the greatest vital debility,—cases in which, the loss of but a moderate quantity of blood, would hurry the patient to his tomb; while this fancied criterion of acute disease, is often, nay commonly, absent in maladies, where severe inflammation is combined with the greatest constitutional strength. In affections, moreover, which require the repeated abstraction of blood, it has often been remarked, that the buff-coat has not appeared on the first or second bleeding, yet on the third or fourth, it has been copiously exhibited. What shall we say, then, of the doctrine of this tunic's characterizing inflammatory action, and warranting the repeated abstraction of blood,—the doctrine, I mean, unlimited and unguarded?" P. 115.

We must now conclude, as our limits are exceeded. Mr. Thackrah's little work, as our readers must perceive, is by far the best which we have on the subject, and entitles its author to the thanks and esteem of his professional brethren.

---

